# Editorial: Endocrine regulation of ovarian follicle development and oocyte maturation: molecular mechanisms and functional insights

**DOI:** 10.3389/fendo.2026.1851500

**Published:** 2026-04-28

**Authors:** Bo Pan, Suranga P. Kodithuwakku

**Affiliations:** 1 The Henry M. Jackson Foundation for the Advancement of Military Medicine (HJF), Bethesda, MD, United States; 2 University of Peradeniya, Peradeniya, Sri Lanka; 3 Institute of Veterinary Medicine and Animal Sciences, Estonian University of Life Sciences, Tartu, Estonia

**Keywords:** assisted reproductive technology (ART), endocrine regulation, folliculogenesis, oocyte competence, polycystic ovary syndrome (PCOS), poor ovarian response (POR)

The development of ovarian follicles and the acquisition of oocyte competence are fundamental determinants of female fertility, shaped by the dynamic interplay between systemic endocrine cues and local intra-ovarian signaling. Dysregulation of these interactions contributes to infertility, poor ovarian response, and complex syndromes such as polycystic ovary syndrome (PCOS). These factors greatly influence clinical outcomes in assisted reproductive technologies (ART), increasing costs and imposing psychological burdens on patients due to treatment failures. This Research Topic was initiated to highlight the molecular mechanisms mediating the endocrine control of folliculogenesis and oocyte maturation. By placing emerging mechanistic findings alongside clinically meaningful evidence, we aim to guide future research and improve patient-centered reproductive care. Across the nine contributions assembled here, three overarching messages emerge. First, endocrine interventions in ART benefit from optimization; however, improvements in intermediate outcomes (e.g., oocyte maturation) do not always translate into higher pregnancy or live birth rates. Second, endocrine regulation of follicular function is closely intertwined with cellular metabolism and oxidative stress, particularly in PCOS and poor-prognosis settings. Third, follicle fate decisions-from primordial activation to sex-specific gonadal differentiation, depend on context-specific receptor networks and pathway crosstalk, revealing potential alternative routes that can compensate when canonical signaling is perturbed.

## Optimizing endocrine strategies in ART: efficacy, safety, and meaningful endpoints

A major translational theme of this topic concerns how endocrine manipulation can be tailored to maximize benefit while minimizing risk. Central to this discussion is the work of Beebeejaun et al. who conducted a systematic review and network meta-analysis to evaluate and rank final oocyte maturation trigger strategies: human chorionic gonadotropin (hCG), GnRH agonist (GnRHa), dual, and double triggers, in predicted high responders undergoing antagonist protocols. Their findings demonstrate that GnRHa, hCG, and dual triggers exhibit comparable efficacy in terms of oocyte yield, oocyte maturity (MII rates), and clinical pregnancy rates. Notably, the GnRHa trigger stands out for its superior safety profile, significantly reducing the risk of moderate-to-severe ovarian hyperstimulation syndrome (OHSS). While these findings provide immediate clinical guidance, they also underscore the importance of balancing efficacy with safety, particularly the risk of OHSS, when selecting trigger strategies.

*In vitro* maturation (IVM) offers a lower-risk alternative to conventional IVF by minimizing or avoiding ovarian stimulation; however, its widespread clinical adoption has been limited by variable success rates and protocol heterogeneity. To address these limitations, Lin et al. synthesized randomized trial evidence evaluating whether FSH priming improves IVM outcomes. Their findings highlight an important translational reality: similar to trigger strategies, endocrine modulation via FSH priming may improve maturation metrics, yet broader reproductive outcomes often remain unchanged. This reflects both protocol variability and the complexity of defining and measuring true “oocyte competence.” While optimizing safety is paramount for high responders, the challenge for low-prognosis patients lies in maximizing efficiency per cycle. The shift from the static definition of “poor ovarian response” to the dynamic POSEIDON (Patient-Oriented Strategies Encompassing Individualized Oocyte Number) criteria, proposed in, 2016, enables more refined patient stratification based on age, ovarian biomarkers, and prior stimulation history, thereby supporting more individualized care. Building on this framework, two original clinical studies provide real-world and longitudinal insights. Deloire et al. reported clinical use patterns and outcomes of Follitropin delta in patients at risk of hypo-response (POSEIDON groups 3 and 4), offering practice-based evidence that complements controlled trials. In parallel, Wei et al. evaluated whether increasing gonadotropin doses in a subsequent antagonist cycle improves cumulative live birth rates in POSEIDON groups 1 and 2, emphasizing the importance of patient-relevant endpoints and cost-benefit considerations rather than assuming that higher stimulation universally leads to better outcomes.

Collectively, these studies reinforce a key principle in modern reproductive endocrinology: treatment individualization should be guided by robust evidence rather than the assumption that more aggressive stimulation is inherently beneficial. Clinical success should be assessed using patient-centered outcomes rather than relying solely on intermediate laboratory metrics.

## Metabolic and endocrine integration in follicular function and ovarian disorders

A second thematic cluster underscores the interdependence of endocrine signaling and metabolic regulation within the follicular microenvironment. Polycystic ovary syndrome (PCOS), the most prevalent endocrine disorder among women of reproductive age, affects approximately 8-13% globally and remains a leading cause of anovulatory infertility. However, its clinical significance extends beyond reproduction, as PCOS is closely linked to long-term metabolic dysfunction, including a substantially increased risk of type 2 diabetes and associated healthcare burden. These observations reinforce the need to consider PCOS as a systemic disorder in which reproductive and metabolic pathophysiology are tightly coupled.

Within this context, from Zheng et al. explored the combined effects of Metformin and Curcumin in a PCOS rat model. Their findings indicate that combination therapy improves endocrine and metabolic parameters more effectively than monotherapy, including restoration of estrous cyclicity, normalization of ovarian morphology, and attenuation of hyperandrogenism and insulin resistance. The observed reductions in oxidative stress markers (ROS, MDA) and enhancement of antioxidant defenses (GPx, SOD, GSH) further support a mechanistic link between metabolic stress and follicular dysfunction. Nevertheless, the translational relevance of these findings remains to be established, particularly given the limitations inherent to preclinical models and the variability of PCOS phenotypes in humans.

At the cellular level, Chen et al. provide evidence that altered lipid metabolism in granulosa cells from PCOS patients is associated with c-Fos–mediated transcriptional regulation. This study highlights a potential interface between steroid receptor signaling and metabolic reprogramming within the follicle, although the causal relationships and downstream functional consequences warrant further clarification. Complementing these findings, Guo et al. conducted a non-targeted metabolomic analysis of follicular fluid in patients with poor ovarian response (POR), identifying 40 differential metabolites enriched in pathways related to glycerophospholipid metabolism, choline metabolism, and autophagy. While the identification of perillyl aldehyde as a candidate biomarker is intriguing, its clinical utility will depend on validation in larger, well-characterized cohorts and its predictive value relative to established markers.

Taken together, these studies support an emerging framework in which endocrine regulation, metabolic pathways, and oxidative homeostasis are tightly interconnected in shaping follicular function and oocyte competence. At the same time, they highlight a persistent gap between mechanistic insight and clinical translation, underscoring the need for integrative, longitudinal, and functionally validated approaches.

## Follicle fate decisions and receptor-dependent developmental routes

A detail study from Gamaleri et al. demonstrates that endocrine regulation extends to early follicle fate decisions and developmental trajectories. Their work explores chemokine receptor signaling in the ovarian cortex and its role in primordial follicle activation, highlighting a potential immune-endocrine interface relevant to follicle recruitment and long-term ovarian reserve dynamics.

Extending beyond mammalian systems, Wang et al. investigated ovarian differentiation in turtles, dissecting the roles of estrogen receptor signaling under different temperature conditions. Their findings illustrate how endocrine pathways can drive ovarian differentiation in specific contexts, while alternative temperature-dependent mechanisms may independently support feminization. These results underscore a broader principle: endocrine outcomes are highly context-dependent and may involve compensatory or parallel pathways conserved across species.

## Final outlook

This Research Topic highlights a shift in reproductive endocrinology toward integrative, mechanism-driven frameworks. A key priority is translating mechanistic insights into clinically actionable tools, particularly robust biomarkers derived from granulosa cell signaling, follicular fluid multi-omics, and endocrine response profiles. Crucially, these markers must be validated against meaningful endpoints such as cumulative live birth rates rather than intermediate outcomes.

Future research should also adopt integrative designs that capture the interplay between endocrine signaling, metabolism, and oxidative stress, especially in heterogeneous conditions such as PCOS and stratified populations defined by frameworks like POSEIDON. In parallel, comparative and cross-species approaches may reveal conserved and alternative regulatory pathways, refining current models of follicle development and supporting more precise, patient-centered reproductive care.

